# Lack of associations between hospital rating and outcomes in patients with an acute coronary syndrome

**DOI:** 10.1136/bmjoq-2023-002475

**Published:** 2024-03-21

**Authors:** Sara Aspberg, Thomas Kahan, Fredrik Johansson

**Affiliations:** 1 Division of Cardiovascular Medicine, Department of Clinical Sciences, Danderyd Hospital, Karolinska Institutet, Stockholm, Sweden; 2 Department of Clinical Sciences, Danderyd Hospital, Karolinska Institutet, Stockholm, Sweden

**Keywords:** healthcare quality improvement, performance measures, hospital medicine, clinical practice guidelines

## Abstract

**Background:**

Public reporting of performance data has become a common tool in evaluation of healthcare providers. The rating may be misleading if the association between the measured variables and the outcome is weak.

**Methods and results:**

Nationwide, register-based, cohort study. All Swedish patients hospitalised with an acute coronary syndrome during the time periods 2006–2010 and 2015–2017 were included in the study. Possible associations between cardiovascular morbidity and mortality for these patients and ranking scores for each hospital in a Swedish healthcare quality register for acute coronary syndromes were analysed. We found no association between the ranking score and mortality, and no or weak associations between the ranking score and readmissions.

**Conclusions:**

Lack of associations between quality measurements and patient outcomes warrants improvement in ranking scores. Cautious use of the ranking results is necessary in comparisons between healthcare providers.

**WHAT IS ALREADY KNOWN ON THIS TOPIC**

- Public comparisons between healthcare providers based on performance data are common but may be misleading.

**WHAT THIS STUDY ADDS**

- We found no or weak associations between hospital rankings based on a Swedish healthcare quality register for patients with coronary heart disease, and mortality and hospitalisation due to acute coronary syndromes.

**HOW THIS STUDY MIGHT AFFECT RESEARCH, PRACTICE OR POLICY**

- Careful interpretation of performance data and relevant outcome measures may improve future evaluation of healthcare providers.

## Introduction

Assessment of quality in healthcare has rendered an increasing interest over the years.^1^ Disease burden and mortality rates have been compared between countries and healthcare systems for decades to help decision-makers to direct their efforts to improve health. Also, public performance data make comparisons possible between hospitals, outpatient clinics or even on an individual physician level. This has been controversial; advocates believe that this strengthens patient power, while antagonists claim that data are misleading unless you are aware of its flaws.^2–4^

Another intention with ranking and public release of performance data is to improve quality in healthcare by competition. By highlighting units with unfavourable quality in healthcare providers must improve, or the patient will abandon them. This assumption implies that quality in healthcare can be defined and measured. However, due to variation in the focus and measures between rating systems, high performance hospitals within one rating system may not be high performers within another rating system. This confuses patients, providers and purchasers, and makes it difficult for hospitals to understand where to focus their efforts for improvement.^5–7^ Further, patients may be unable or unwilling to use hospital rankings in decisions regarding choice of healthcare providers. The Swedish healthcare system, based on public financing, may also limit the importance of competition between healthcare providers.

Healthcare quality registries have become a common tool for evaluations of hospital performance. There is a belief that mere participation in clinical registries increases quality by the feedback registries provide. It is, however, difficult to separate the impact of quality registries from an underlying trend. Of note, two studies comparing outcome measures as mortality, readmission rates and complications for hospitals participating in the American College of Surgeons National Surgical Quality Improvement Programme with hospitals that did not, found that participation did not render any additional improvements in quality beyond an underlying positive trend.^8 9^ Other studies provide support for the usefulness of participation in registries, for example, for the choice by informed patients of high-quality hospitals and for associations between adherence to guidelines-based therapy for acute coronary syndromes (ACS) and lower mortality.^10–12^

Sweden has several nationwide healthcare quality registries of high standard. Some of them are used for publicly ratings of hospitals. Among these is the Swedeheart registry, including patients with an ACS.^13^ Swedeheart has several goals, including supporting adherence to guidelines, improvements of evidence-based therapies, and reduction of mortality and morbidity. Cardiovascular care in Swedish hospitals is publicly rated by Swedeheart since 2006.

The aim of this study was to test the accuracy of the Swedish hospital rating system. We used results from the Swedeheart registry and assessed possible correlations between rating scores and data on morbidity and mortality at a hospital level, per year and over time.

## Methods

### Data sources

#### Swedeheart

The Register of Information and Knowledge about Swedish Heart Intensive care Admissions (RIKS-HIA) is a registry dedicated to the care of patients with an ACS. It started as a regional registry in 1991 and has developed gradually. Since 2008 RIKS-HIA covers all hospitals in Sweden taking care of patients with an ACS. The registry aims to improve cardiac care of patients with coronary heart disease by continuous reporting on treatment regimens and results.

**Figure 1:**
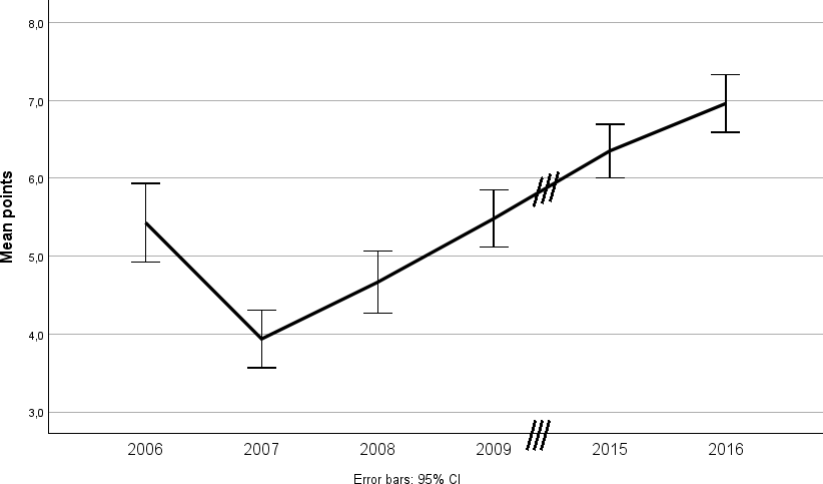
Mean ratings per year.

To facilitate comparisons of hospitals a quality index was developed. The first public ranking of hospitals was published in 2006, based on the results in 2005. In the first ranking, the hospitals were not disclosed by name, but since 2007 also the names of the hospitals are revealed.^14^ The quality index originally included nine process measures, all of high priority when it comes to high quality care of an ACS. Each measure could render 0, 0.5 or 1 point depending on the degree of fulfilment rendering a score of maximum 9 points. During the studied period, the required levels for received points were revised; hence the ranking score a following year could be the same or lower despite better performance. This is illustrated in figure 1, showing trends in mean ratings, with a steep decline in ratings between 2006 and 2007. The decline is not explained by a sudden drop in adherence to guideline recommended therapy. Instead, the required levels for some of the process measures were increased, for example, from inclusion of 70% to 80% of eligible patients. Changes in guideline recommendations could also change the point score, such as the lower recommended target levels of LDL-cholesterol, from initially below 3.0, to below 2.5, 1.8 and now below 1.4 mmol/L. The point score for target LDL-cholesterol values was adjusted following these recommendations. Due to a large variation between hospitals regarding patients older than 80 years admitted to cardiac care units, and a lack of evidence-based treatments in this age group, patients older than 80 were excluded in the quality index.

In 2009, RIKS-HIA merged into Swedeheart together with three other healthcare quality registries within cardiology, SEcondary Prevention after Heart Intensive care Admissions, Swedish Coronary Angiography and Angioplasty Registry and the Swedish Heart Surgery Registry. The RIKS-HIA quality index underwent a major revision in 2011 and developed into the Swedeheart quality index, now including outcome measures and gradually also patients older than 80 years, with the aim to improve the registry coverage and to better reflect secondary prevention (table 1).

**Table 1:** Measures used in the rating system before and after 2011

Before 2011	After 2011
Reperfusion for STEMI/LBBB	Reperfusion for STEMI/LBBB
PCI as primary reperfusion for STEMI/LBBB (2006) Reperfusion for STEMI/LBBB within recommended time (2007–2009)	Reperfusion for STEMI/LBBB within recommended time
Coronary angiography in target group with NSTEMI	Coronary angiography in target group with NSTEMI
LMW heparin/heparin/fondaparinux during care event or PCI performed within 24 hours for NSTEMI	–
Aspirin or other thrombocyte inhibitor or anticoagulants at discharge following myocardial infarction	–
P2Y12-receptor blocker for NSTEMI	P2Y12-receptor blocker for NSTEMI
Beta-blocker at discharge following myocardial infarction.	–
Lipid-lowering drugs at discharge for myocardial infarction.	–
ACE-inhibitor/ARB in target group for myocardial infarction	ACE-inhibitor/ARB in target group for myocardial infarction
–	Share with myocardial infarction as main diagnosis (<80 years) included in RIKS-HIA
–	Share of myocardial infarctions <75 in RIKS-HIA undergoing follow-up (SEPHIA)
–	Share of smokers that had stopped smoking after 12–14 months
–	Share that had participated in exercise programme after 12–14 months
–	Share with LDL-cholesterol<2.5 mmol/L (96.7 mg/dL) after 12–14 months
–	Share with systolic blood pressure <140 mm Hg after 12–14 months

ARB, angiotensin receptor blocker; LBBB, left bundle branch block; LDL, low density lipids; LMW, low molecular weight; NSTEMI, non-ST elevation myocardial infarction; PCI, percutaneous coronary intervention; RIKS-HIA, Register of Information and Knowledge about Swedish Heart Intensive care Admissions; SEPHIA, SEcondary Prevention after Heart Intensive care Admissions; STEMI, ST elevation myocardial infarction.

The score could now reach a maximum of 11 points. The construction of the quality index in year 2016 is given in table 2 as an example.

**Table 2:** Points per measurement in 2016

Quality measurement	0.5 point, %	1 point, %
Reperfusion in STEMI/LBBB	80	85
Reperfusion in STEMI/LBBB within recommended time	75	90
Coronary angiography in NSTEMI	75	80
P2Y12-blockers in NSTEMI	85	90
ACEI/ARB in myocardial infarction, target group	85	90
Proportion of patients with myocardial infarction as major diagnosis (<80 years)	90	95
Proportion of patients with myocardial infarction as major diagnosis (<75 years) undergoing follow-up	70	90
Proportion of smokers who have stopped smoking 12–14 months after the myocardial infarction	60	70
Proportion of patients who have participated in physical training programme 12–14 months after the myocardial infarction	50	60
Proportion of patients who have LDL cholesterol <1,8 mmol/L or >50% reduction 12–14 months after the myocardial infarction	40	60
Proportion of patients with systolic blood pressure <140 mm Hg 12–14 months after the myocardial infarction	70	75

ARB, angiotensin receptor blocker; LBBB, left bundle branch block; LDL, low density lipids; NSTEMI, non-ST elevation myocardial infarction; STEMI, ST elevation myocardial infarction.

Due to the years chosen for analysis in this study, we used both the original RIKS-HIA quality index and the Swedeheart quality index as up to 2016. The ranking scores are presented in yearly published reports from Swedeheart.^15^

#### The Swedish National Patient Register

Patients admitted for ACS were identified through the Swedish National Patient Register. All publicly financed hospitals in Sweden are required to report basic information about hospital admissions, including main and secondary discharge diagnoses, to the National Patient Register.^16^ There are no privately financed hospitals in Sweden offering care for patients with an ACS. A validity study of the register has previously shown that data is of high standard, for definite acute myocardial infarction the diagnosis was correct in 95% of the cases.^17^

#### The cause of death register

The national Cause of Death Register includes the date and supposed underlying cause of death among Swedish citizens and has a completeness of 100%.^18^ This register was used to calculate mortality rates at 30 and 365 days.

### Defining hospital units

There is a hospital mismatch between the National Patient Register and Swedeheart, as a hospital could be regarded as one single unit in the National Patient Register but counted as several units in Swedeheart. In the National Patient Register, four hospitals counted as two separate units each, and two hospitals counted as four separate units by Swedeheart. These hospitals were given a rating score weighted by number of patients at their units in Swedeheart, as outcomes by hospital were not available (online supplemental table 1).

10.1136/bmjoq-2023-002475.supp1 Supplementary data

### Study cohort

All patients admitted to a Swedeheart ranked hospital with an ACS from 1 January 2006 to 31 December 2009 and from 1 January 2015 to 31 December 2016 were included. The first time period was chosen since these years were the first in public reporting of ACS healthcare performance in Sweden, representing a transition time in healthcare evaluation. The second time period was chosen to represent a time period when the new Swedeheart quality index introduced in 2011, was established after some years of development and changes. An ACS was defined as a main discharge diagnosis in the National Patient Register with any of the International Classification of Diseases, tenth revision (ICD-10) codes I20.0, I21.0–9 or I22.0–9. No ICD-9 codes were used since Sweden shifted from ICD-9 to ICD-10 in 1997. The code for unstable angina pectoris (I20.0), but no other diagnosis for angina pectoris was included as we aimed to evaluate ACS.

### Outcome definitions

Outcomes were defined as hospital readmission due to an ACS, as a marker for coronary artery disease and all-cause mortality. Readmissions were identified from the National Patient Register with a main ICD-10 code of acute myocardial infarction (I21.0–I21.9), reinfarction (I22.0–I22.9) or unstable angina pectoris (I20.0). The ACS was assumed to occur on the day of admission. To exclude false readmission due to transfer between hospitals readmission had to take place 30 days after primary admission date. Few patients have hospital stays exceeding 30 days. Only the first admission for each patient for 1 year was included in the analysis. Mortality rates at 30 days and at 365 days after the discharge date were calculated for each hospital. The analyses were repeated for each year during the study period, which implies that the same patient could be included several times in separate analyses.

#### Patient and public involvement

Patients or public were not actively involved in the work with this study.

### Statistical analysis

Descriptive statistics are given as numbers, proportions and rates. Rates are given in percent at 30 and 365 days. Differences between rates 2006 and the following years were analysed with analysis of variance (ANOVA), where a probability (p)<0.05 was regarded as significant. Associations between the individual hospital ranking scores, and readmissions and mortality for the same year were first analysed with linear regression, based on Pearson’s correlation analysis due to the assumed interval character of the scale. In the next step, Spearman’s correlation analysis aimed for ordinal scales was used to detect possible, non-linear correlations. The readmission analysis was adjusted for the competing risk of death by stratifying patients into groups based on survival.

We finally added a linear regression model where adjustment for age was included.

IBM SPSS Statistics V.22 (IBM, USA) was used for all statistical analyses.

## Results

A total of 69 hospitals defined by the National Patient Register were rated by Swedeheart. These 69 hospitals encompass all hospitals in Sweden offering care for patients with an ACS. The quality index score per year and hospital is given in online supplemental table 1.

**Figure 2:**
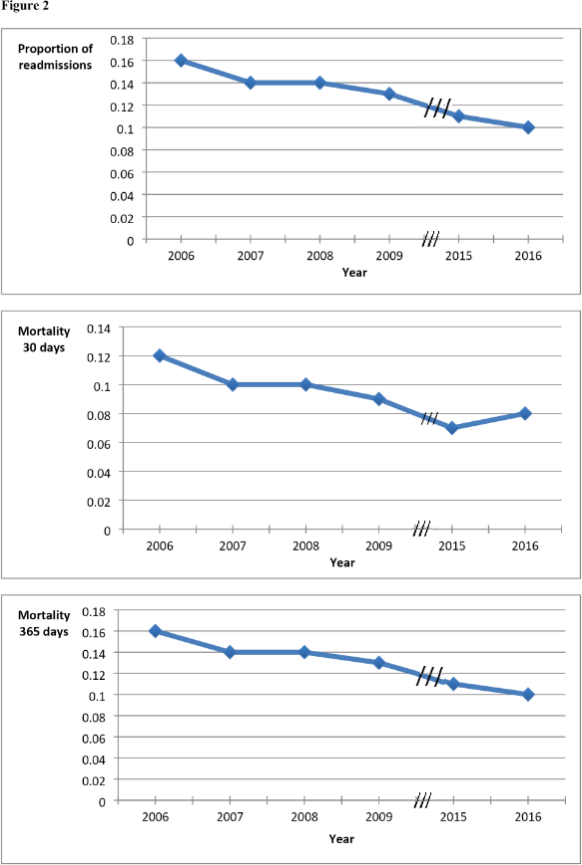
Mortality and readmissions per year.

We identified unique patients with an ACS in the National Patient Register for each year during study time. The total number of patients per year decreased during the period, reflecting a decrease in the incidence of ACS (online supplemental table 2). Readmission and mortality rates also decreased during study time (figure 2). The decrease in readmission rate was not significant between 2007 and 2008 (p=0.56) but significant between all other years analysed. The mortality rates followed a similar pattern. There was a trend (p=0.055) for a decrease in 1-year mortality between 2007 and 2008, a highly significant (p<0.001) decrease in 30-day and 1-year mortality between 2008 and 2009, but no decrease in mortality between 2015 and 2016.

10.1136/bmjoq-2023-002475.supp2 Supplementary data

**Figure 3:**
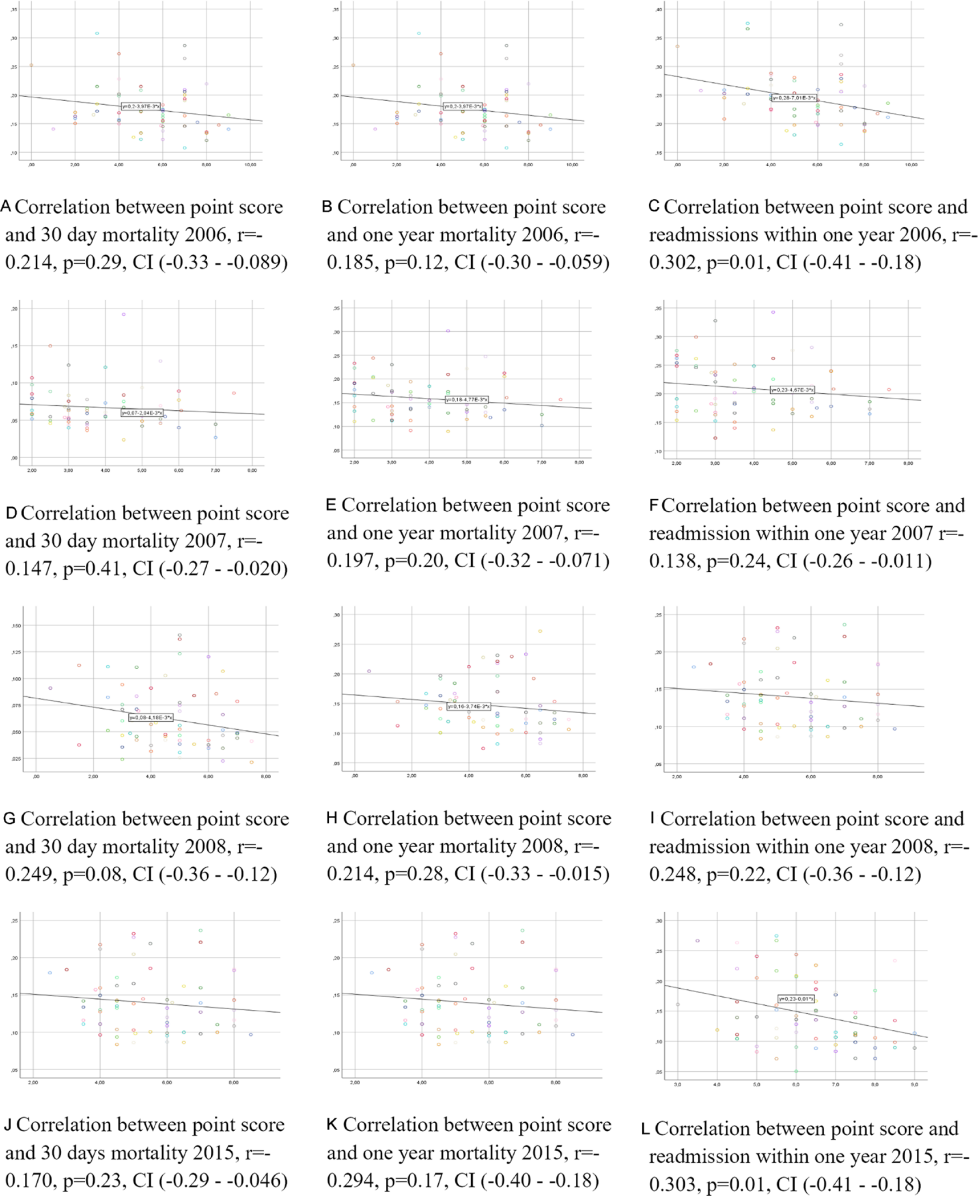
Correlations between ranking scores, mortality and readmissions.

Possible associations between the Swedeheart quality index and readmission and mortality rate per year are presented with Pearson’s correlation coefficients in figure 3. There were no correlations between the quality index and mortality rate for any year included in the study. Furthermore, there were no correlations between the quality index and readmission rates, except for readmissions 2006 (r=−0.302, p=0.010) and 2015 (r=−0.303, p=0.010) based on Pearson’s correlation analysis. The non-parametric analyses, adjusting for the competing risk of death by stratification, gave similar results with a correlation between the quality index and readmissions only in 2006 (r=0.326, p=0.009). The multivariable regression model including adjustment for age did not change the overall results, except for an association between the point score and readmission in 2015 (β=−0.301, p=0.017), but no associations between the point score and readmissions during previous years.

## Discussion

This study evaluates the association between hospital rating and cardiovascular readmissions and mortality rates based on the national Swedeheart registry of ACS. The assessments were performed both at transition to public rating of hospitals and some years later, after modification of the rating score. We found no correlations between the quality index and mortality rates at a hospital level. Furthermore, we found no or weak correlations between the quality index and readmission rates. The incidence of ACS decreased during the study period, but our results do not suggest an association between the ranking score and mortality and readmission rates for patients hospitalised for ACS.

Variations in hospital provision of guideline recommended treatment were previously reported to explain 28% of the between-hospital variation in 30 days mortality in patients with an acute myocardial infarction included in Swedeheart 2004–2011, after adjustments for casemix.^12^ In the same report, variation in hospital treatment explained 22% of the variation in 30 days mortality after acute myocardial infarction in patients included in the Myocardial Ischaemia National Audit Project (MINAP) in the UK. This association is high compared with a study in USA, where only 6% of hospital-level variation in 30 days mortality rates for patients with an acute myocardial infarction was explained by public reported process measures.^19^ The discrepancy in associations between Swedeheart and MINAP may at least for the first study period be explained by the coverage for these registries. By the time of the study, both registries covered only 50%–60% of all patients hospitalised for an acute myocardial infarction, and the included patients were younger and less diseased, whereas we included every patient with ACS as primary diagnosis at discharge.^20 21^ The modified Swedeheart index (used from 2011) includes the degree of coverage, that is, the share of patients eligible for inclusion in the registry who is included, as a part of the index. However, this modification did not impact the lack of association between the score and cardiovascular events after an ACS. Another explanation might be that patients with ST elevation myocardial infarction (STEMI) are over-represented in the RIKS-HIA/Swedeheart population. A study of the association between in-hospital mortality rates and a composite index of performance measures found a weak significant association for STEMI but not for non-STEMI patients.^22^ For RIKS-HIA/Swedeheart, the selection bias is in part explained by the fact that some hospitals only register patients treated in cardiac intensive care units, which was also the original intention of the registry. Of note, the indication for admission to these units (or to a cardiology unit without intensive care facilities) varies between hospitals, especially for elderly patients.

Hospital characteristics and socioeconomic status are other factors not examined in our study, which explain variations in mortality rate between hospitals. A multivariable model including hospital characteristics and socioeconomic status explained 17% of the variation in hospital-specific 30 days risk-stratified mortality rates after an acute myocardial infarction.^23^ This notwithstanding, most of the variation in mortality rates between hospitals remains unexplained and warrant further study. Given that most of the variation in cardiovascular outcome may be explained by other factors than current in-hospital treatment regimens, it is reasonable to believe that hospitals in general have limited opportunities to deviate from secular mortality trends. This said severe non-adherence to guideline-based treatments is likely to result in adverse deviation from secular mortality trends.

In the worst-case scenario, such associations may even be reversed. Settings where public rating is linked to economical reimbursement may create an incentive to avoid taking care of patients with perceived high risk of worse outcome.^24^ Higher ratings have under such circumstances been linked to higher mortality.^25^

The weak associations between the quality index and readmission rates are in line with a larger study including 2700 hospitals in the USA, were the association between performance measures and 30 days all-cause readmission rate after acute myocardial infarction was low (r=0.10, although significant). Other evaluated conditions in that study (pneumonia and orthopaedic surgery) also had low associations (r=0.07 and 0.06, respectively) between performance measures and readmission rate.^26^

On average, hospitals increased their rating scores during the study period. This suggests improved performance measures, a higher quality of care, or maybe differences in reporting of the quality measures or the way the score was calculated. As hospitals achieve a higher degree of fulfilment regarding process measures, the differences between them will diminish. Although this is a good thing, the association between performance measure and outcome on a hospital level will be weaker when all hospitals have a high compliance to guidelines. Also, the differences in rating score in Swedeheart are disproportionately large, as compared with treatment regimen, as points are given by reaching a threshold value rather than by use of a continuous scale. Hence, the quality index may exaggerate the relative differences in treatments between hospitals. Another reason for weak associations between performance measures and outcome at a hospital level might be that each hospital has too few cases, rendering a large variability in mortality and readmission rate per hospital.

There are important limitations of this study. First, although the National Patient Register has an almost complete degree of coverage of ACS there is scarce information about risk factors and other potentially important confounding factors for prognosis for the individual patient. Thus, the comparisons are based on crude and partly unadjusted data. However, the use of risk-adjusted mortality rates to evaluate healthcare is also associated with pitfalls.^27^ Further, the Swedeheart quality index is not based on adjusted patient data, and case mix in different hospitals is not taken into account. Thus, we consider the crude analysis reflecting the way the quality index is used. However, the year-wise, cross-sectional analyses in the study do not allow us to follow patients over a longer time, nor to follow changes in the rating system.

Second, during the first part of the study period, RIKS-HIA/Swedeheart was focused on patients aged 80 years or less treated in coronary care or intensive care units. We included all patients with an ACS as main diagnosis at discharge on purpose, which included also patients older than 80 years of age and those hospitalised outside cardiac care units. We consider this to give a higher degree of coverage and a better reflection of the quality of ACS care for the individual hospital, and hence more useful comparison between hospitals.

Third, readmissions and mortality after discharge from hospital are influenced by the follow-up of the patient. Although Sweden has a rather standardised way of follow-up during the first year after an ACS, differences in follow-up will impact the correlations. This may be of special importance for elderly patients or patients with many comorbidities.

### Conclusion

Mortality rates and readmission rates in patients hospitalised for an ACS associate poorly to a Swedish registry based index specifically evaluating quality of care at a hospital level. Ranking scores based on process measures might be one important dimension to better understand, evaluate and improve hospital healthcare. However, the use of ranking scores as a useful support for patient decision-making and to enhance quality improvement remains to be established. Further studies, with focus on finding process measures and quality indicators associated to relevant outcomes, may help us to develop and improve the quality scores.

10.1136/bmjoq-2023-002475.supp3 Supplementary data

## Data Availability

Data are available on reasonable request. The data underlying this article will be shared on reasonable request to the corresponding author.

## References

[1] Porter ME , Larsson S , Lee TH . Standardizing patient outcomes measurement. N Engl J Med 2016;374:504–6. 10.1056/NEJMp1511701 26863351

[2] Werner RM , Bradlow ET . Public reporting on hospital process improvements is linked to better patient outcomes. Health Affairs 2010;29:1319–24. 10.1377/hlthaff.2008.0770 20606180

[3] Werner RM , Asch DA . The unintended consequences of publicly reporting quality information. JAMA 2005;293:1239–44. 10.1001/jama.293.10.1239 15755946

[4] Wang DE , Wadhera RK , Bhatt DL . Association of Rankings with cardiovascular outcomes at top-ranked hospitals vs Nonranked hospitals in the United States. JAMA Cardiol 2018;3:1222–5. 10.1001/jamacardio.2018.3951 30484836 PMC6583101

[5] Austin JM , Jha AK , Romano PS , et al . National hospital ratings systems share few common scores and may generate confusion instead of clarity. Health Affairs 2015;34:423–30. 10.1377/hlthaff.2014.0201 25732492

[6] Raghuram AC , Dasari TK , Chou B , et al . Confusion instead of clarity: publicly reported cardiac surgery ratings for coronary artery bypass Grafting and aortic valve replacement. J Am Coll Surg 2019;228:180–7. 10.1016/j.jamcollsurg.2018.07.663 30359838

[7] Hota B , Webb T , Chatrathi A , et al . Disagreement between hospital rating systems: measuring the correlation of multiple benchmarks and developing a quality composite rank. Am J Med Qual 2020;35:222–30. 10.1177/1062860619860250 31253048

[8] Etzioni DA , Wasif N , Dueck AC , et al . Association of hospital participation in a surgical outcomes monitoring program with inpatient complications and mortality. JAMA 2015;313:505–11. 10.1001/jama.2015.90 25647206

[9] Osborne NH , Nicholas LH , Ryan AM , et al . Association of hospital participation in a quality reporting program with surgical outcomes and expenditures for Medicare beneficiaries. JAMA 2015;313:496. 10.1001/jama.2015.25 25647205 PMC4337802

[10] Kaye DR , Norton EC , Ellimoottil C , et al . Understanding the relationship between the centers for Medicare and Medicaid services' hospital compare star rating, surgical case volume, and short-term outcomes after major cancer surgery. Cancer 2017;123:4259–67. 10.1002/cncr.30866 28665483 PMC5650526

[11] Shah BR , O’Brien EC , Roe MT , et al . The Association of in-hospital guideline adherence and longitudinal post discharge mortality in older patients with non-ST-segment elevation myocardial infarction. Am Heart J 2015;170:273–80. 10.1016/j.ahj.2015.05.007 26299224

[12] Chung S-C , Sundström J , Gale CP , et al . Comparison of hospital variation in acute myocardial infarction care and outcome between Sweden and United kingdom: population based cohort study using nationwide clinical registries. BMJ 2015;351:h3913. 10.1136/bmj.h3913 26254445 PMC4528190

[13] Highlights och Kvalitetsindex 2023. 2022 Available: https://www.ucr.uu.se/swedeheart/

[14] Årsrapport 2006 - RIKS-HIA och SEPHIA. 2022 Available: https://www.ucr.uu.se/swedeheart/dokument-rikshia/arsrapporter-rikshia/arsrapport-2006-riks-hia-och-sephia/

[15] . 2024 Available: https://www.ucr.uu.se/swedeheart/dokument-sh/arsrapporter-sh/arsrapporter-sh-aldre

[16] Statistical database, diagnoses. 2022 Available: https://sdb.socialstyrelsen.se/if_par/val_eng.aspx

[17] Rosén M , Alfredsson L , Hammar N , et al . Attack rate, mortality and case fatality for acute myocardial infarction in Sweden during 1987-95. results from the National AMI register in Sweden. J Intern Med 2000;248:159–64. 10.1046/j.1365-2796.2000.00716.x 10947895

[18] Statistical database, cause of death. n.d. Available: https://sdb.socialstyrelsen.se/if_dor/val_eng.aspx

[19] Bradley EH , Herrin J , Elbel B , et al . Hospital quality for acute myocardial infarction: correlation among process measures and relationship with short-term mortality. JAMA 2006;296:72–8. 10.1001/jama.296.1.72 16820549

[20] Aspberg S , Stenestrand U , Köster M , et al . Large differences between patients with acute myocardial infarction included in two Swedish health registers. Scand J Public Health 2013;41:637–43. 10.1177/1403494813483936 23567645

[21] Herrett E , Shah AD , Boggon R , et al . Completeness and diagnostic validity of recording acute myocardial infarction events in primary care, hospital care, disease Registry, and national mortality records: cohort study. BMJ 2013;346:f2350. 10.1136/bmj.f2350 23692896 PMC3898411

[22] Kontos MC , Rennyson SL , Chen AY , et al . The Association of myocardial infarction process of care measures and in-hospital mortality: a report from the NCDR. Am Heart J 2014;168:766–75. 10.1016/j.ahj.2014.07.005 25440806

[23] Bradley EH , Herrin J , Curry L , et al . Variation in hospital mortality rates for patients with acute myocardial infarction. Am J Cardiol 2010;106:1108–12. 10.1016/j.amjcard.2010.06.014 20920648

[24] Blumenthal DM , Valsdottir LR , Zhao Y , et al . A survey of Interventional Cardiologists’ attitudes and beliefs about public reporting of percutaneous coronary intervention. JAMA Cardiol 2018;3:629–34. 10.1001/jamacardio.2018.1095 29801157 PMC6145664

[25] McCabe JM , Waldo SW , Kennedy KF , et al . Treatment and outcomes of acute myocardial infarction complicated by shock after public reporting policy changes in New York. JAMA Cardiol 2016;1:648. 10.1001/jamacardio.2016.1806 27463734

[26] Stefan MS , Pekow PS , Nsa W , et al . Hospital performance measures and 30-day readmission rates. J Gen Intern Med 2013;28:377–85. 10.1007/s11606-012-2229-8 23070655 PMC3579957

[27] Goodacre S , Campbell M , Carter A . What do hospital mortality rates tell us about quality of care Emerg Med J 2015;32:244–7. 10.1136/emermed-2013-203022 24064042

