# Supplementary material for: Lack of associations between hospital rating and outcomes in patients with an acute coronary syndrome
Source: BMJ Open Qual. 2024 Mar 21;13(1):e002475. doi: 10.1136/bmjoq-2023-002475 (PMC10961561; doi:10.1136/bmjoq-2023-002475)
Supplement: Supplementary data [file bmjoq-2023-002475supp001.pdf]

Suppl Table 1. Hospitals with ranking score

Hospital	Ranking score per year					
	**2006**	**2007**	**2008**	**2009**	**2015**	**2016**
Akademiska sjukhuset	9	7	6	6	6.5	8.0
Alingsås lasarett	4	2	4	5.5	5	6
Arvika sjukhus	3	4.5	3.5	7	6.5	6.5
Avesta lasarett	5	3	4	4	5	5
Blekingesjukhuset[1]	4.7	2	4.2	5.0	--	--
- Karlshamn[1]	4	2	4.5	3.5	6.5	5
- Karlskrona[1]	5	2	4	6	4.5	7.5
Bollnäs sjukhus	6	4	5	6.5	8	6.5
Danderyds sjukhus	6	3	6	8	8.5	9
Enköpings lasarett	7	5	6.5	6	4.5	5.0
Falu lasarett	6	2.5	3	4.5	6	7.5
Gällivare	--	--	--	--	5.5	7.5
Gävle sjukhus	6	6	5	6	8	8
Hallandssjukhus Halmstad	4	3	5	3.5	5	5.5
HallandssjukhusVarberg	7	3	4.5	5	6.0	6.5
Helsingborgs lasarett	7	3	4	4.5	6	5
Huddinge sjukhus	8	4	6.5	7	6.5	7.0
Hudiksvalls sjukhus	4	5	4.5	5	5.5	8
Hässleholms sjukhus	4	2.5	3	5	6	5.5
Höglandssjukhuset	4	2	5	7	6	6
Kalix lasarett	4	2.5	1.5	4	6	5.5

Karlskoga lasarett	6	5	5	5	5	8
Karlstads sjukhus	8	4.5	5.5	5.5	7	6
Karolinska sjukhuset	8	3.5	6	5	6.5	8.0
Kiruna lasarett	3	2	2.5	3.5	5.5	7
Kristianstads sjukhus	6	5	6.5	6	4.5	7
Kullbergska sjukhuset	2	5.5	7.5	7.5	6.5	9
Kungälvs sjukhus	7	3.5	3.5	3.5	7	9.5
Köpings lasarett	7	3	5	4.5	9	8.5
Lindesbergs lasarett	5	3	5	3	4.5	6.5
Ljungby lasarett	6	5.5	5	6	6.5	7.5
Lycksele lasarett	7	3.5	4	4	3	4
Länssjukhuset Kalmar	6	4	3	4.5	6	7
Mora lasarett	2	4	6.5	7	5.5	6.5
Motala lasarett	7	6	4.5	5	6.5	7
Mälarsjukhuset	5	7	7	7.5	7.5	7.5
Norrlands Universitetssjukhus	7	3.5	5	4	8	8.5
Norrtälje sjukhus	3	2.5	3	6.5	6.5	8.5
NU-sjukvården[2]	5	2	3.3	4.5	7	6.5
- Trollhättan[2]	--	--	4	--	--	--
- Uddevalla[2]	--	--	2	--	--	--
- Borås[2]	9	6.5	7	8	4.0	4.5
- Skene[2]	7	5	3.5	5	--	--
Nyköpings lasarett	4	5.5	7.5	8	8.5	8.5
Oskarshamns sjukhus	6	4.5	6	6	7	8

Piteå	--	--	--	--	5.5	6.0
Sahlgrenska Universitetssjukhuset[3]	5.9	2.9	3.6	3.9	5.5	5.8
- Mölndals sjukhus[3]	4	2	2.5	3.5	--	--
- Cardiac intensive care unit[3]	6	3.5	4	5	--	--
- Medical emergency department[3]	--	2.5	4	--	--	--
- Östra sjukhuset[3]	7	3	3.5	4	--	--
Skaraborgs sjukhus[4]	2.8	2.9	3.3	6.2	5.5	6.0
- Lidköping[4]	5	4	3	6.5	--	--
- Skövde[4]	2	2.5	3.5	6	--	--
Skellefteå lasarett	0	3.5	3.5	4.5	3.5	2
Sollefteå sjukhus	2	3	0.5	4	7	8.5
S:t Görans sjukhus[5]	7.6	5.6	7.5	8.5	7.5	8.5
- Cardiac intensive care unit[5]	8	6	--	--	--	--
- Chest pain center[5]	6	3.5	--	--	--	--
Sunderbyn	--	--	--	--	5	8.5
Sundsvalls sjukhus	7	4	5	6	6	7
Södersjukhuset	7	3.5	6	7	8.5	9
Södertälje sjukhus	5	2.5	3	4	4.5	6.5
Torsby sjukhus	1	3.5	3.5	8	6.5	6.5
Trelleborgs lasarett	7	6	6.5	6.5	7	8.5
Universitetssjukhuset i Linköping[6]	6	7.5	7	5.3	7.5	7.5
- Cardiac intensive care unit[6]	6	7.5	7	6	--	--
- Medical emergency department[6]	--	--	--	3	--	--
Universitetssjukhuset i Lund	7	3	5	6	7.5	8.5

Universitetssjukhuset MAS	8	5.5	7	6	7.5	8.5
Universitetssjukhuset i Örebro	5	5	4.5	4.5	7.5	8.5
Visby lasarett	3	2	3	4	4.5	4.5
Vrinnevisjukhuset	4	3	5	5.5	6	7.5
Värnamo sjukhus	5	4.5	5.5	7	6.5	5.5
Västerås lasarett	7	7	6.5	8	9	9.5
Växjö lasarett	6	4.5	5	4.5	8.5	7
Ystads lasarett	4	2	2.5	2.5	7.5	6
Ängelholms sjukhus	6	2	1.5	4	5.5	4.5
Örnsköldsviks sjukhus	5	4.5	3.5	3.5	6	6.5
Östersunds sjukhus	2	2	3	5	4.5	7

[1] Counts as one unit in the National Patient Register (Blekingesjukhuset) and as two units in Swedeheart (Karlskrona and Karlshamn)

[2] Counts as one unit in the National Patient Register (NU-sjukvården) and as four units in Swedeheart (Trollhättan, Borås, Uddevalla, and Skene)

[3] Counts as one unit in the National Patient Register (Sahlgrenska Universitetssjukhuset) and as four units in Swedeheart (Cardiac intensive care unit, Medical emergency department, Mölndal, and Östra)

[4] Counts as one unit in the National Patient Register (Skaraborgs sjukhus) and as two units in Swedeheart (Lidköping and Skövde)

[5] Counts as one unit in the National Patient Register (S:t Görans sjukhus) and as two units in Swedeheart (Cardiac intensive care unit and Chest pain center)

[6] Counts as one unit in the National Patient Register (Universitetssjukhuset i Linköping) and as two units in Swedeheart (Cardiac intensive care unit and Medical emergency department)
