# Supplementary material for: Lack of associations between hospital rating and outcomes in patients with an acute coronary syndrome
Source: BMJ Open Qual. 2024 Mar 21;13(1):e002475. doi: 10.1136/bmjoq-2023-002475 (PMC10961561; doi:10.1136/bmjoq-2023-002475)
Supplement: Supplementary data [file bmjoq-2023-002475supp002.pdf]

**Suppl Table 2** Patients with acute coronary syndromes, per year

| **Year** | **Patients (number)** |
|---|---|
| 2006 | 29,682 |
| 2007 | 29,478 |
| 2008 | 28,175 |
| 2009 | 26,369 |
| 2015 | 22,436 |
| 2016 | 21,186 |
